# HOW TO TRAIN ENOUGH PROFESSIONAL DEMENTIA CAREGIVERS IN CHINA TO MEET THE FAST GROWING NEED?

**DOI:** 10.1093/geroni/igae098.0229

**Published:** 2024-12-31

**Authors:** Zengliang Gong, Gege Cai, Chunjuan Xu

**Affiliations:** Taikang Community, Beijing, Beijing, China; Taikang Community, Beijing, Beijing, China; Taikang Community, Beijing, Beijing, China

## Abstract

**Purpose:**

This paper try to establish an effective system for training dementia caregivers basing on the practices of a leading provider in China. Method: 1. Establish a holistic memory care program and curriculum system, and deliver them through a set of training programs to cover both first-line caregivers and managers, such as an 8-hour orientation session for newcomers, 23-hours on-job training per year, 40-hour training for new managers. 2. Estabilish a case competition among all communities of the provider across China, so that the team could learn from the specific care practices and from each other. 3. Test different training programs empirically to improve training effectiveness.

**Results:**

1. The memory care team in the provider has grown substantically, and the number of certified trainee of 8-hour orientation session has increased to 1400 since 2021. 2. The turnover rate of caregivers in the memory care area is 5% lower than the overall turnover rate of the care building. The turnover rate for memory care managers dropped from 57% in 2021 to 21% in 2023. 3. The experimental group that learned the concept and values of “person-centered care” first was significantly better than the control group in learning knowledge and skills in dementia care.

**Conclusion:**

Systematic and practice-based training programs are conducive to quickly cultivating professional caregivers for dementia care. The Chinese government and the senior care industry should invest more resources in establishing similar training programs to meet the urgent needs of people with dementia and their families.

